# NLRP3 Inflammasome Contributes to Host Defense Against *Talaromyces marneffei* Infection

**DOI:** 10.3389/fimmu.2021.760095

**Published:** 2021-11-29

**Authors:** Haiyan Ma, Jasper F. W. Chan, Yen Pei Tan, Lin Kui, Chi-Ching Tsang, Steven L. C. Pei, Yu-Lung Lau, Patrick C. Y. Woo, Pamela P. Lee

**Affiliations:** ^1^ Department of Pediatrics and Adolescent Medicine, Li Ka Shing Faculty of Medicine, The University of Hong Kong, Hong Kong, Hong Kong SAR, China; ^2^ Department of Microbiology, Li Ka Shing Faculty of Medicine, The University of Hong Kong, Hong Kong, Hong Kong SAR, China

**Keywords:** *Talaromyce marneffei*, dectin-1, caspase-1, NLRP3 inflammasome, ASC, CD4 T cells

## Abstract

*Talaromyce marneffei* is an important thermally dimorphic pathogen causing disseminated mycoses in immunocompromised individuals in southeast Asia. Previous studies have suggested that NLRP3 inflammasome plays a critical role in antifungal immunity. However, the mechanism underlying the role of NLRP3 inflammasome activation in host defense against *T. marneffei* remains unclear. We show that *T. marneffei* yeasts but not conidia induce potent IL-1β production. The IL-1β response to *T. marneffei* yeasts is differently regulated in different cell types; *T. marneffei* yeasts alone are able to induce IL-1β production in human PBMCs and monocytes, whereas LPS priming is essential for IL-1β response to yeasts. We also find that Dectin-1/Syk signaling pathway mediates pro-IL-1β production, and NLRP3-ASC-caspase-1 inflammasome is assembled to trigger the processing of pro-IL-1β into IL-1β. *In vivo*, mice deficient in NLRP3 or caspase-1 exhibit higher mortality rate and fungal load compared to wild-type mice after systemic *T. marneffei* infection, which correlates with the diminished recruitment of CD4 T cells into granulomas in knockout mice. Thus, our study first demonstrates that NLRP3 inflammasome contributes to host defense against *T. marneffei* infection.

## Introduction

*Talaromyce marneffei* is an opportunistic dimorphic pathogenic fungus that causes fatal systemic mycosis in immunocompromised hosts in southeast Asia (1). It is considered as an AIDS-defining disease ranking the third most common opportunistic infection in HIV-positive patients following tuberculosis and cryptococcosis in endemic areas (1–3). In recent years, with the improvement of antiretroviral therapy and the effective control of the HIV/AIDS epidemic, the incident cases are increasingly reported in HIV-negative patients with transplantation, autoimmune diseases and primary immunodeficiency diseases (4–9).

*T. marneffei* is thermally dimorphic; it grows as a mold bearing conidia when cultured at 25°C, whereas it converts into yeast cells at 37°C (3). It is believed that airborne *T. marneffei* conidia are inhaled by patients from the surrounding environment. Upon inhalation, conidia can adhere to pulmonary epithelial cells *via* interaction with laminin and fibronectin on the cell membrane, and then directly transform into yeast cells (10, 11). Subsequently, yeast cells are phagocytosed by pulmonary macrophages. The proliferating yeast cells within macrophages trigger granuloma formation, followed by the dissemination of yeast cells into other organs, such as the liver, spleen, skin, *via* bloodstream (3, 12).

Immune responses against fungal infection are mounted by fungal pathogen-associated molecular patterns (PAMPs) that can be recognized by pathogen recognition receptors (PRRs), including Toll-like receptors (TLRs), C-type lectin receptors (CLRs), and NOD-like receptors (NLRs) (13). NLRP3 is a member of the NLR family, and it is able to indirectly sense danger signals, e.g. PAMPs (14). Upon activation, NLRP3 interacts with adaptor protein ASC that can further recruit pro-caspase-1, leading to the assembly of multiprotein complex known as the NLRP3 inflammasome (15). Thereafter, pro-caspase-1 can be activated through auto-proteolysis, resulting in the release of caspase-1 p10 and p20 (14). Active caspase-1 not only processes pro-IL-1β and pro-IL-18 into bioactive IL-1β and IL-18, but also mediates pyroptosis (16).

It has been well established that inflammasome activation plays critical roles in the control of fungal infections, including those caused by *C. albicans* and *A. fumigatus* (17, 18). It is found that Dectin-1 and TLR2 are engaged in the production of pro-IL-1β in response to *C. albicans*, and NLRP3, ASC and caspase-1 control the processing of IL-1β to defend against disseminated fungal infection and mortality *in vivo* (17). In a murine model of disseminated candidiasis, the NLRP3 inflammasome is suggested to exert antifungal host defense through driving protective Th1 and Th17 immune responses (19). Furthermore, in response to *Aspergillus* infection, NLRP3 in collaboration with AIM2 mediates the inflammasome activation through ROS generation and potassium efflux, and both caspase-1 and caspase-8 are associated with processing of IL-1β and IL-18 (18). Subsequently, IL-1β and IL-18 production regulates the effector function of CD4 T cells (primarily Th17 and Th1 cells) that are crucial for controlling disseminated fungal infection (19, 20). However, to date, whether NLRP3 inflammasome activation contributes to host defense against *T. marneffei* is still unknown.

In this study, we investigated whether and how *T. marneffei* induces NLRP3 inflammasome activation and whether the activated inflammasome has a protective role in defense against *T. marneffei* infection. Using *in vitro* and *in vivo* models, we found that *T. marneffei* yeasts rather than conidia induce a robust IL-1β response in human PBMCs. IL-1β release in response to yeast cells varies among different cell types. The Dectin-1/Syk signaling pathway mediates pro-IL-1β production, and the NLRP3 inflammasome is activated to enhance the processing of pro-IL-1β and pro-IL-18 into mature IL-1β and IL-18 in response to yeasts. NLRP3 inflammasome could confer protection against systemic *T. marneffei* infection.

## Methods and Materials

### Fungi

*T. marneffei and C. albicans* were isolated from patients by the Department of Microbiology, Queen Mary Hospital. *T. marneffei* conidia were cultured on Sabouraud Dextrose Agar (SDA) plates at 25°C for 7 days, and then collected in sterile water by wet cotton swabs, washed 3 times and re-suspended in sterile water, and finally filtered through 40µm nylon filters. To prepare *T. marneffei* yeasts, *T. marneffei* conidia were cultured on SDA plates at 37°C for 10 days, and then collected in sterile 0.1% PBST by wet cotton swabs, washed 3 times and re-suspended in RPMI 1640 with shaking for 24 hr at 37°C, and finally filtered through 40µm nylon filters. *C. albicans* was cultured on SDA plates at 30°C for 48hr, and then transferred to Sabouraud Dextrose (SD) broth and incubated at 37°C with shaking for 16–24hr to obtain the yeast form of *C. albicans*. At the same time *C. albicans* was cultured in enriched media consisting of RPMI+10% fetal bovine serum (FBS) at 37°C for 24h to obtain the pseudo-hyphae form of *C. albicans*. For fixed fungal preparation, fungi were washed in sterile PBS, fixed in 4% paraformaldehyde (PFA) solution for 10 min, and then washed three times in sterile PBS. For heat-killed fungal preparation, fungi were boiled at 100°C for 40 min.

### Mice

*Nlrp3^-/-^* mice (B6.129S6-Nlrp3tm1Bhk/J) and *Casp1^-/-^* mice (B6.129S2-Casp1tm1- Flv/J) were purchased from the Jackson Laboratory. Wild-type C57BL/6 (WT) mice were offered by the Laboratory Animal Unit (LAU) at the University of Hong Kong. All mice were bred in the specific-pathogen-free animal facilities at the Minimal Diseased Area (MDA) of LAU, the University of Hong Kong. In all mice experiments described here, male and female mice aged 8-12 weeks were used. All mouse experiments were performed in compliance with institutional guidelines for the use of experimental animals and the protocols approved by the Committee on the Use of Live Animals in Teaching and Research (CULATR) at the University of Hong Kong.

### Cell Isolation and Culture

Peripheral blood mononuclear cells (PBMCs) were isolated from the buffy coats of healthy donors according to the principles of the Declaration of Helsinki and were approved by the responsible ethics committee (Institutional Review Boards of the University of Hong Kong/Hospital Authority Hong Kong West Cluster). Human primary CD14^+^ monocytes were isolated from PBMCs by magnetic labeling with CD14, followed by positive selection with MACS columns and separators (Miltenyi Biotec). For isolation of monocyte-derived macrophages, PBMCs were cultured in 10cm dish, and medium was changed gently at 2hr after cell seeding. Cells were left overnight, and adherent cells were treated with cold 5mM EDTA for 10 min, scrapped and cultured in 24-well plates for 12 days. During this period, medium was changed on day 7 and day 9 to remove non-adherent cells. Human PBMCs, CD14^+^ monocytes, monocyte-derived macrophages were cultured in RPMI 1640 supplemented with 5% heat-inactivated autologous sera and 1% penicillin/streptomycin. For monocyte-derived dendritic cells (DCs), CD14^+^ monocytes were cultured for 5 days in RPMI1640 containing 10% FBS, 1% penicillin/streptomycin, human IL-4 (40ng/ml, PeproTech) and GM-CSF (50ng/ml, PeproTech). Non-adherent or loosely adherent cells were collected.

Murine bone marrow-derived dendritic cells (BMDCs) were prepared as previously described (21). Briefly, bone marrow cells were isolated from the femurs and tibias of mice. Red blood cells were lysed on ice for 5 min by using 1x RBC Lysis Buffer (BioLegend, 420301). Bone marrow cells (3x10^6^ per well) were cultured in 6-well plates in 3 ml of complete medium (RPMI 1640, 1% penicillin/streptomycin, 10% FBS, 1% Glutamax, 1% sodium pyruvate, and 55μM β-mercaptoethanol) supplemented with murine GM-CSF (20 ng/ml, Peprotech). Half of the medium was removed on day 2 and fresh medium supplemented with murine GM-CSF (40 ng/ml) was added. The culture medium was entirely aspirated on day 3 and replaced by fresh medium containing GM-CSF (20 ng/ml). Non-adherent cells in the culture supernatant and loosely adherent cells were harvested.

### Cell Stimulation

PBMCs (4x10^6^/ml) were co-cultured with live, PFA-treated or heat-killed *T. marneffei*, as well as live, heat-killed or PFA-treated *C. albicans* yeasts or pseudohyphae at 0.5MOI for indicated time points. PBMCs (4x10^6^/ml), monocytes (2x10^6^/ml), monocyte-derived macrophages (1x10^6^/ml), or monocyte-derived DCs (1x10^6^/ml) were primed with LPS (10ng/ml for PBMCs and monocytes, 100ng/ml for monocyte-derived macrophages and monocyte-derived DCs, *Invivo*Gen) for 3hr and then stimulated with heat-killed *T. marneffei* yeasts at 0.5 MOI for 18hr. Human CD14^+^monocytes (2x10^6^/ml) were pre-incubated with human anti-Dectin-1 (10μg/ml, *Invivo*Gen), anti-TLR2 blocking antibodies (10μg/ml, *Invivo*Gen), or corresponding isotype controls (10μg/ml, *Invivo*Gen), or different concentrations of R406 (*Invivo*Gen), or Z-YVAD(OMe)-FMK (ALX-260-074, Enzo Life Sciences) for 1hr before co-culture with heat-killed *T. marneffei* yeasts. Murine BMDCs (2x10^6^/ml) were primed with or without LPS (100 ng/ml) for 3hr, and then co-cultured with heat-killed *T. marneffei* yeasts at 1 MOI for additional 18 hr. Alternatively, Nigericin (10 μM) was added into LPS-primed cells in the last 40 min for positive control.

### Cytokine Assays

Cytokine concentration in culture supernatant was measured by commercial ELISA kits: human IL-1β (ebioscience), human TNF-α, IL-18, and IFN-γ, IL-17A (R&D systems), and murine IL-1β, TNF-α (R&D systems) according to manufacturer’s instructions. IL-6, MCP-1, IL-8, IL-18 and IFN-γ from human PBMCs were measured by LEGENDplex™ Human Inflammation Panel (Biolegend) according to manufacturer’s instructions.

### Western Blot

Total cell lysates were prepared using sample buffer containing 5% β-mercaptoethanol. Protein concentration was determined by bicinchoninic acid (BCA) assay. Concentrated culture supernatant was prepared as previously described (22). Briefly, cell culture supernatant was precipitated by adding an equal volume of methanol and 0.25 volumes of chloroform. It was vigorously vortexed and centrifuged at 20,000 g for 10 min, and the upper phase was aspirated and discarded, and 500 μl methanol was added and centrifuged at 20,000 g for 10 min. Then protein pellet was dried in 55°C water baths for 5 min. Proteins from cell lysates (20-50 μg) or concentrated supernatant were boiled, separated on SDS-PAGE, and transferred onto PVDF membranes (Bio-Rad Laboratories). Membranes were blocked with 5% bovine serum albumin (BSA) in Tris-buffered saline (TBS) with 0.1% Tween 20 for 1hr at room temperature, incubated overnight at 4°C with the following primary antibodies diluted 1:1000 in 5% BSA/TBST: anti-pro-IL-1β (#12703, Cell Signaling Technology) and anti-NLRP3 (AG-20B-0014-C100, Adipogen Life Sciences), and anti-mouse caspase-1 (AG-20B-0042-C100, Adipogen Life Sciences), and then incubated for 1hr with goat anti-rabbit and goat anti-mouse secondary antibodies, respectively, at room temperature. Membranes were washed and exposed by adding enhanced chemiluminescence (ECL) in dark room. When necessary, stained membranes were incubated in stripping buffer at 65°C for 10 min, and washed 3 times with 0.1% TBS-Tween 20, and blocked with 5% BSA for 1hr, and stained with anti- β-actin rabbit mAb (4970S, Cell Signaling Technology) followed by goat anti-rabbit secondary antibody staining and film exposure.

### Confocal Imaging of ASC Specks

Murine BMDCs (2x10^6^/ml) were seeded on the chamber slides (154941, Nalge Nunc International) and co-cultured with heat-killed *T. marneffei* yeasts at 1 MOI for 18 hr. BMDCs were fixed with 4% PFA for 20 min and permeabilized with 0.1% Triton-X-100 for 10 min. Cells were washed 3 times and blocked with 5% BSA for 1hr, followed by incubation with anti-ASC rabbit polyclonal antibody (SC-22514-R, Santa Cruz Biotechnology) overnight at 4°C. After washing, cells were incubated with AlexaFlour-488 conjugated goat anti-rabbit secondary antibody (Life Technologies) at room temperature for 1 hr. Subsequently, cells were stained with Alexa Fluor 647 phalloidin (ThermoFisher Scientific, A22287) for 30 min at room temperature avoiding light. Finally, slides with cells were mounted with ProLong Gold Antifade Mountant with DAPI (Invitrogen) and analyzed by confocal microscopy (Zeiss LSM 710). For quantification of ASC speck, 40-150 monocytes per field under microscopy with magnification 40x were manually analyzed, and the ratio of ASC speck positive cells to total cells was calculated. At least 3 fields per sample were counted.

### Flow Cytometry

To detect Syk phosphorylation, CD14^+^ monocytes (2x10^6^/ml) were co-cultured with heat-killed *T. marneffei* yeasts at 0.5 MOI for 18hr. Cells were fixed with BD Phosflow Fix Buffer I for 10 min at 37°C, followed by permeabilization with BD Phosflow Perm Buffer III for 30 min on ice. After washing, cells were stained with PE-conjugated phospho-Syk (Tyk525/526) antibody (#6485, Cell Signaling Technology) for 30 min on ice, and analyzed by flow cytometer (LSR II, BD).

### Murine Systemic Talaromycosis Model

For *in vivo T. marneffei* yeasts infection, WT, *Nlrp3* ^-/-^, *Casp1* ^-/-^ mice were intravenously injected with 100 μl of suspension containing 5x10^5^ colony forming units (CFUs) live *T. marneffei* yeasts in sterile PBS. Mice survival was daily monitored following infection and mice were sacrificed once they showed signs of the humane endpoints. In the second batch of infection experiments, mice were sacrificed, and spleens and livers were harvested at 7 or 14 days post infection (dpi) of *T. marneffei* yeasts. Part of spleen and liver were weighed and homogenized in sterile PBS, and a series of diluted solutions of cell suspensions were plated onto SDA plates. Fungal load was assessed after culturing for 2 days at 25°C. Part of spleen and liver were fixed in 4% paraformaldehyde (PFA) and paraffin slides were prepared for hematoxylin and esosin (HE) staining and Grocott’s methenamine silver (GMS) staining. The stained sections were mounted and observed under microscope for histopathological assessment. For CD4 T cells labeling by immunohistochemistry (IHC), liver sections were incubated overnight with primary antibodies, anti-CD4 antibody (ab183685, Abcam) after blocking the endogenous peroxidase and non-specific binding with hydrogen peroxide and 10% normal goat serum, respectively. Then biotin-conjugated goat anti-rabbit IgG were added, and sections were sequentially incubated with HRP conjugated streptavidin and DAB, and finally counterstained with hematoxylin. For quantification of CD4 positive cells in liver, the number of CD4 positive cells were counted in randomly selected 10 granulomas per section.

### Statistical Analysis

Statistical analysis was carried out using GraphPad Prism v6.0 software. Data were represented as mean ± SEM. Statistical significance was determined by two-tailed Student’s t test, one-way ANOVA or two-way ANOVA with multiple comparison tests, log-rank test for comparison of survival rate. p<0.05 was considered statistically significant.

## Results

### *T. marneffei* Yeasts, but Not Conidia, Induced Potent IL-1β Response in Human PBMCs

**Figure 1:**
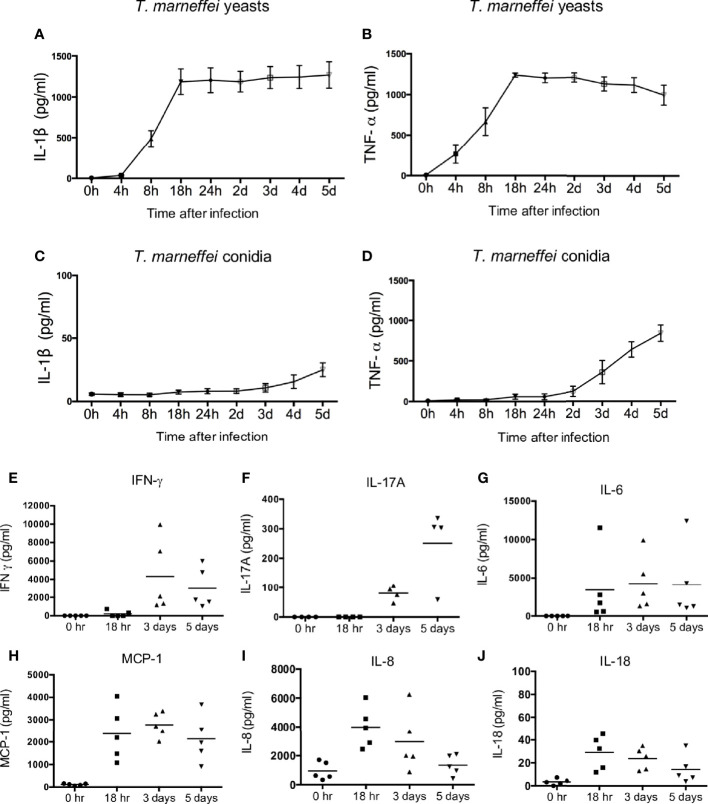
*T. marneffei* yeasts, but not conidia, induce potent IL-1β response in human PBMCs. **(A, B)** Quantification of IL-1β and TNF-α by ELISA in human PBMCs (4x10^6^/ml) infected with live *T. marneffei* yeasts (0.5 MOI) for indicated time points (n=5, mean ± SEM). **(C, D)** ELISA detection of IL-1β and TNF-α in human PBMCs (4x10^6^/ml) infected with live *T. marneffei* conidia (0.5 MOI) for indicated time points (n=5, mean ± SEM). **(E)** Quantification of IFN-γ by LEGENDplex™ in human PBMCs (4x10^6^/ml) infected with live *T. marneffei* yeasts (0.5 MOI) for indicated time points (n=5). **(F)** Quantification of IL-17A by ELISA in human PBMCs (4x10^6^/ml) stimulated with heat-killed *T. marneffei* yeasts (0.5 MOI) for indicated time points (n=4). **(G‒J)** Quantitative analyses of cytokines by LEGENDplex™ in human PBMCs (4x10^6^/ml) infected with live *T. marneffei* yeasts (0.5 MOI) for indicated time points (n=5). “h” and “d” denote “hours” and “days” **(A‒D)**, respectively. Each symbol represents one healthy donor **(E‒J)**.

To dissect the production of cytokines elicited by *T. marneffei*, freshly isolated human peripheral blood mononuclear cells (PBMCs) were co-cultured with *T. marneffei* yeasts or conidia at 0.5 multiplicity of infection (MOI) for indicated incubation time points. As shown in **Figure 1A**, IL-1β became detectable at 8 hr, peaked at 18 hr, and remained plateaued thereafter till day 5 post infection in the culture supernatant of human PBMCs infected with live *T. marneffei* yeasts. Similarly, TNF-α production was detectable as early as 4 hr, and rapidly peaked at 18 hr, and subsequently showed a slight falling trend up to day 5 post infection (**Figure 1B**). These data demonstrated that *T. marneffei* yeasts were an inducer of IL-1β and TNF-a production. In contrast to *T. marneffei* yeasts, only minimal levels of IL-1β was detected in PBMCs co-cultured with conidia at 5 days post infection (**Figure 1C**), whereas TNF-α production began to increase steadily after 2 days of incubation (**Figure 1D**). This suggests that *T. marneffei* conidia were able to trigger TNF-α production in PBMCs but failed to induce IL-1β response.

To examine whether the viability of *T. marneffei* is a prerequisite for the induction of IL-1β in PBMCs, yeasts and conidia were inactivated by heat (100°C for 40 min) or 4% paraformaldehyde (PFA) treatment for 10 min. Our results showed that there was no significant difference in IL-1β production in PBMCs infected with live, heat-killed or PFA-treated *T. marneffei* yeasts (**Figure S1A**), indicating that the viability of *T. marneffei* yeasts was not necessary for the induction of IL-1β response. In contrast, IL-1β production in the PBMCs stimulated with heat-killed conidia was significantly higher compared to live and PFA-treated conidia (**Figure S1B**), suggesting that heat treatment might promote the exposure of some pro-inflammatory PAMPs on the conidia cell wall to induce IL-1β response. In addition, both yeast and pseudo-hyphal forms of *C. albicans* were capable of inducing IL-1β production in human PBMCs, and no differential IL-1β response was observed for heat- and PFA-treated *C. albicans* (**Figure S1C, D**).

We further evaluated the production of other pro-inflammatory cytokines in *T. marneffei* yeasts-infected human PBMCs. As shown in **Figures 1E–J**, IFN-γ and IL-17A could be detected only after 3 days of co-culture, whereas IL-6, MCP-1, IL-8 and IL-18 were detectable as early as 18 hr. Specifically, IFN-γ production reached its maximum at 3 days and slightly reduced at 5 days, while IL-17A production greatly increased from 3 days to 5 days of co-culture (**Figures 1E, F**). IL-6 and MCP-1 production peaked at 18 hr and remained at similar levels at 3 days and 5 days of co-culture (**Figures 1G, H**). IL-8 and IL-18 production was maximal at 18 hr and showed a downward trend thereafter (**Figures 1I, J**). These results suggest that *T. marneffei* yeasts cause the release of cytokines that are associated with innate and adaptive lymphocyte responses.

### Differential Requirement for IL-1β Response to *T. marneffei* Yeasts in Different Human Cell Types

**Figure 2:**
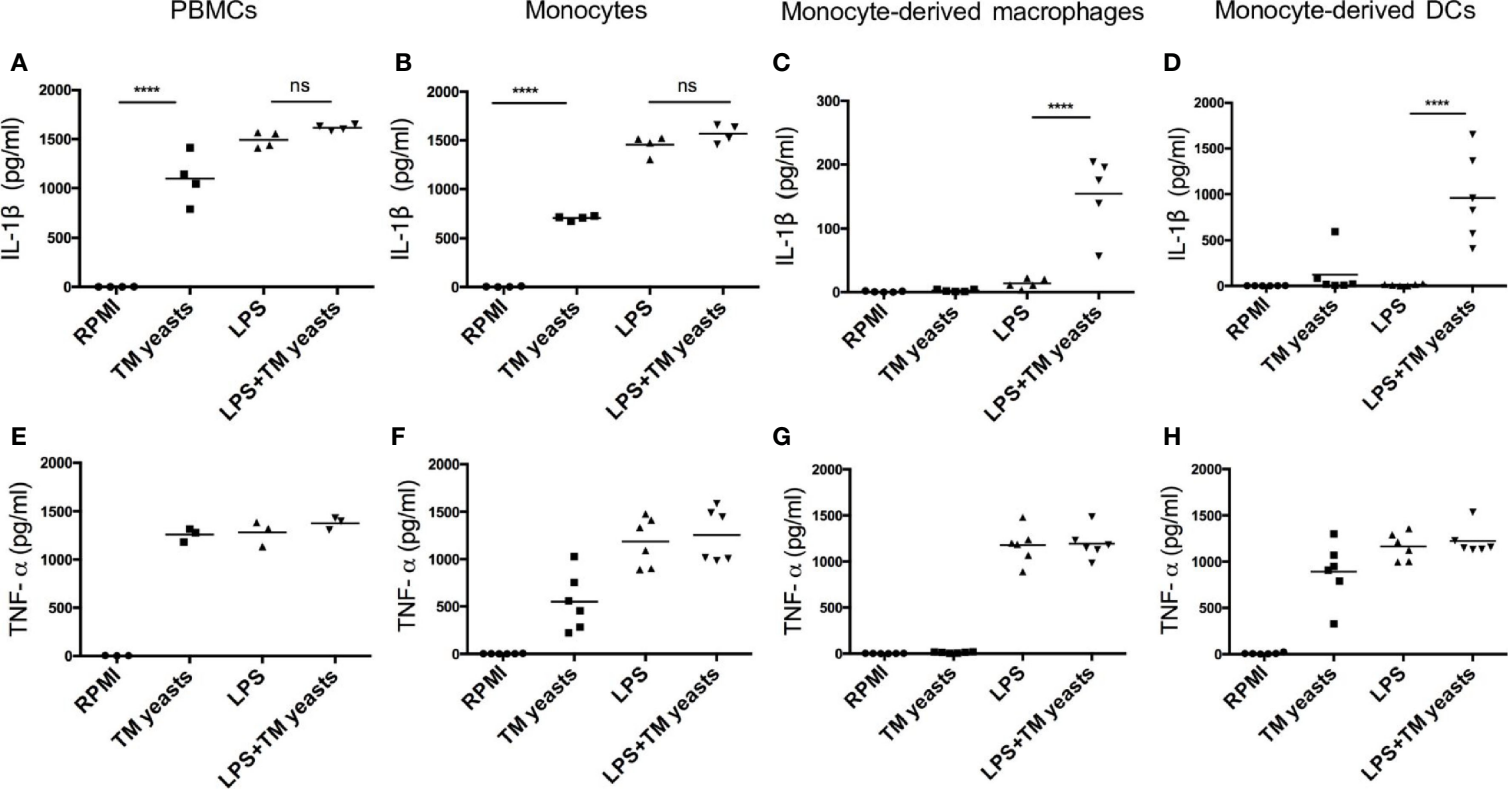
Differential requirement for IL-1β response to *T. marneffei* yeasts in various human immune cell types. **(A‒D)** Quantitative ELISA detection of IL-1β in human PBMCs (4x10^6^/ml), CD14^+^ monocytes (2x10^6^/ml), human monocytes-derived macrophages (1x10^6^/ml), CD14^+^ monocytes-derived dendritic cells (DCs) (1x10^6^/ml), respectively, after stimulation with heat-killed *T. marneffei* (TM) yeasts (0.5 MOI) for 18 hr with or without LPS priming. **(E‒H)** Quantification of TNF-α by ELISA in human PBMCs (4x10^6^/ml), CD14^+^ monocytes (2x10^6^/ml), human monocytes-derived macrophages (1x10^6^/ml), CD14^+^ monocytes-derived dendritic cells (DCs) (1x10^6^/ml), respectively, after stimulation with heat-killed *T. marneffei* (TM) yeasts (0.5 MOI) for 18 hr with or without LPS priming. Each symbol represents one healthy donor, and data are analyzed by one-way ANOVA. ns, not significant, ****p < 0.0001.

It has been suggested that IL-1β production can be mediated by inflammasome activation which requires two signals: the triggering of pro-IL-1β production (signal 1) and the processing of pro-IL-1β into mature IL-1β (signal 2) (15), and IL-1β production from inflammasome activation in response to TLR ligands is differentially regulated in human monocytes and macrophages (23, 24). To investigate whether there was any differential requirement for IL-1β response to yeasts in different cell types, human PBMCs, monocytes, monocytes-derived macrophages and monocytes-derived DCs were primed with or without lipopolysaccharide (LPS) prior to *T. marneffei* yeasts stimulation. As shown in **Figures 2A, B**, *T. marneffei* yeasts alone were able to induce abundant IL-1β production in PBMCs and monocytes, indicating that yeasts provided ‘signal 1’ and ‘signal 2’ for inflammasome activation in these cell types. In contrast, LPS priming was required for IL-1β response to *T. marneffei* yeasts in macrophages and DCs (**Figures 2C, D**). Furthermore, high level of TNF-α could be detected in human PBMCs (**Figure 2E**), monocytes (**Figure 2F**) and DCs (**Figure 2H**), but not in macrophages (**Figure 2G**), after *T. marneffei* yeast stimulation.

### Dectin-1/Syk Signaling Mediated IL-1β Response to *T. marneffei* Yeasts in Human Monocytes

**Figure 3:**
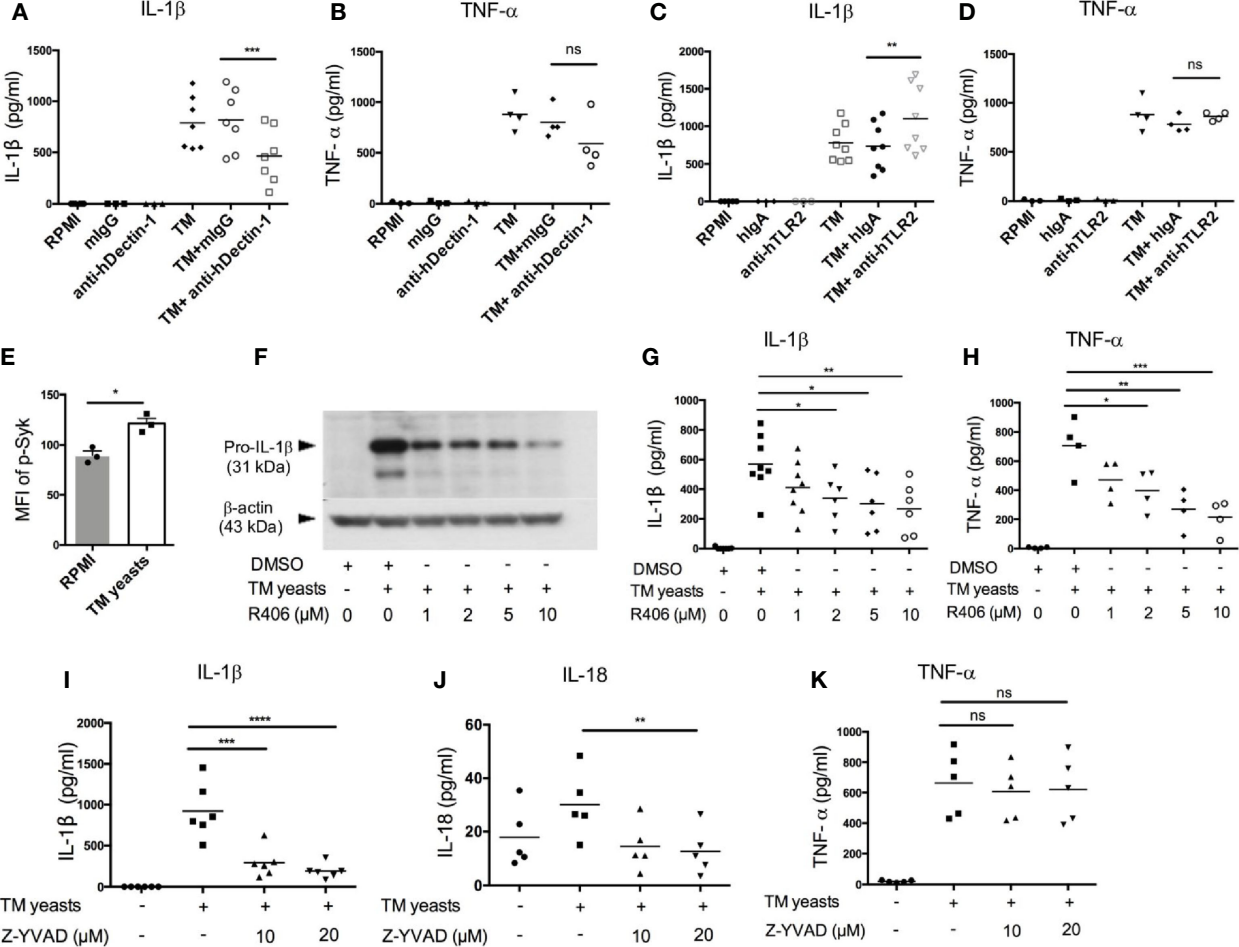
Dectin-1/Syk signaling mediates IL-1β response to *T. marneffei* yeasts in human monocytes. **(A, B)** Quantification of IL-1β and TNF-α by ELISA in human CD14^+^ monocytes (2x10^6^/ml) stimulated with heat-killed *T. marneffei* (TM) yeasts (0.5 MOI) for 18 hr in the presence of anti-hDectin-1 blocking antibodies or mouse IgG (mIgG) (10 μg/ml). **(C, D)** Quantification of IL-1β and TNF-α by ELISA in human CD14^+^ monocytes (2x10^6^/ml) stimulated with heat-killed *T. marneffei* (TM) yeasts (0.5 MOI) for 18 hr in the presence of anti-hTLR2 blocking antibodies or human IgA (hIgA) (10 μg/ml). **(E)** Detection of median fluorescence intensity (MFI) of phospho-Syk by flow cytometry in human CD14^+^ monocytes (2x10^6^/ml) stimulated with heat-killed *T. marneffei* (TM) yeasts (0.5 MOI) for 18 hr (n=3, mean ± SEM). **(F)** A representative immunoblot of pro-IL-β of cell lysates in human CD14^+^ monocytes (2x10^6^/ml) stimulated with heat-killed *T. marneffei* (TM) yeasts (0.5 MOI) for 18 hr in the absence or presence of Syk inhibitor, R406 (n=3). **(G, H)** ELISA quantification of IL-1β and TNF-α in the culture supernatant of human CD14^+^ monocytes (2x10^6^/ml) stimulated with heat-killed *T. marneffei* (TM) yeasts (0.5 MOI) for 18 hr in the absence or presence of Syk inhibitor, R406. **(I‒K)** Quantification of IL-1β, IL-18 and TNF-α by ELISA in the culture supernatant of human CD14^+^ monocytes (2x10^6^/ml) stimulated with heat-killed *T. marneffei* yeasts (0.5 MOI) for 18 hr in the absence or presence of Z-YVAD (caspase-1 inhibitor). Data are analyzed by paired two-tailed t test **(A‒E)** or by one-way ANOVA (G-K). Each symbol represents one healthy donor. ns, not significant, *p < 0.05, **p < 0.01, ***p < 0.001, ****p < 0.0001.

Dectin-1 recognizes β-1,3-glucans in fungal cell wall, which leads to pro-inflammatory immune responses (25). To elucidate the involvement of Dectin-1 in the initiation of IL-1β response to *T. marneffei* yeasts, human CD14^+^ monocytes were co-cultured with heat-killed *T. marneffei* yeasts in the presence of anti-human Dectin-1 or TLR2 neutralizing antibodies or isotype controls (mouse IgG or human IgA). As shown in **Figure 3A**, Dectin-1 blockade resulted in a partial reduction of IL-1β in *T. marneffei* yeasts-stimulated monocytes compared with mouse IgG treatment group. TNF-α production was slightly reduced by Dectin-1 blockade, though not significantly different (**Figure 3B**). These data suggested that Dectin-1 mediated the production of IL-1β in response to *T. marneffei* yeasts in human monocytes. Conversely, blockade of TLR2 resulted in elevated IL-1β secretion and had no effect on TNF-α production in response to yeasts (**Figures 3C, D**), suggesting that TLR2 was not required for enhanced IL-1β response to *T. marneffei* stimulation.

To study the role of Dectin-1/Syk signaling pathway in the induction of IL-1β response to *T. marneffei* yeasts, we measured Syk phosphorylation in *T. marneffei* stimulated human CD14^+^ monocytes by flow cytometry. As expected, *T. marneffei* yeasts induced Syk phosphorylation compared to the unstimulated cells (**Figure 3E** and **Figure S2A**). Pre-treatment of CD14^+^ monocytes with R406 (Syk inhibitor) prior to *T. marneffei* yeasts stimulation resulted in significantly reduced expression of pro-IL-1β in cell lysates (**Figure 3F**) as well as IL-1β in culture supernatant (**Figure 3G**), in a dose-dependent manner. A similar trend of dose-dependent inhibition of TNF-α production in monocytes co-cultured with *T. marneffei* yeasts was also observed (**Figure 3H**). Collectively, our data suggested that Syk, acting as a downstream molecule of Dectin-1 in the initiation of antifungal immunity (25), played an important role in eliciting IL-1β response to *T. marneffei* yeasts in human monocytes.

To verify our hypothesis that caspase-1 activation mediates IL-β and IL-18 release in human monocytes infected with *T. marneffei* yeasts, we pre-treated human CD14^+^ monocytes with caspase-1 inhibitor (Z-YVAD, 10µM and 20µM) prior to co-culture with heat-killed *T. marneffei* yeasts. IL-1β and IL-18 secretion was significantly attenuated in Z-YVAD-treated monocytes (**Figures 3I, J**), whereas inhibition of caspase-1 activity had no obvious effect on TNF-α production (**Figure 3K**), demonstrating that caspase-1 activation contributed to IL-1β and IL-18 release in response to *T. marneffei*. Additionally, we also found that blocking of caspase-1 activation resulted in significantly reduced release of adaptive cytokines, IFN-γ and IL-17A, in response to *T. marneffei* yeasts (**Figure S2B**)

### *T. marneffei* Yeasts Triggered IL-1β Production *via* the NLRP3 Inflammasome

**Figure 4:**
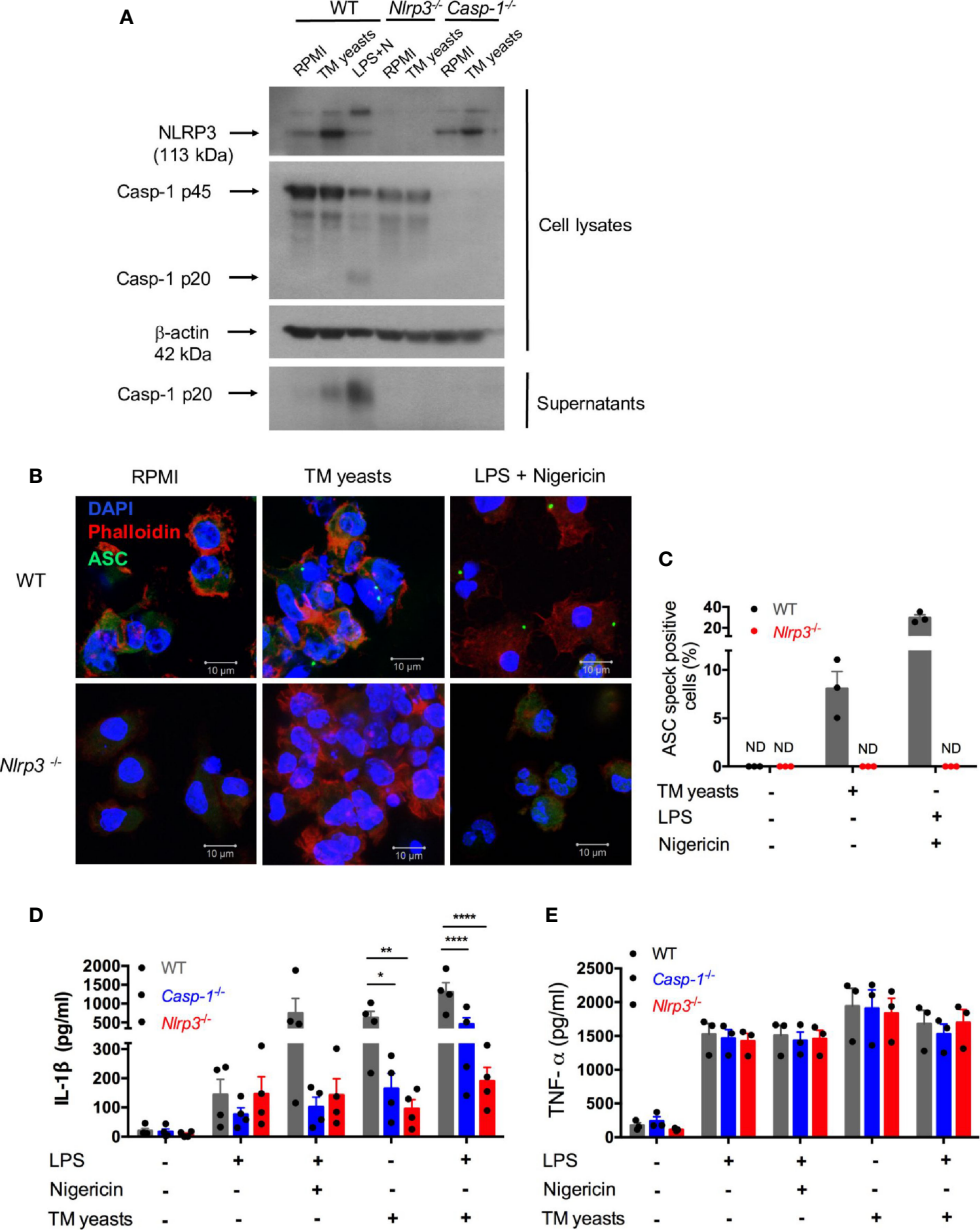
*T. marneffei* yeasts trigger IL-1β production *via* the NLRP3 inflammasome. **(A)** Representative immunoblots of NLRP3, caspase-1 p45 and p20 from cell lysates and caspase-1 p20 from concentrated cell supernatant in WT, *Nlrp3* ^-/-^ and *Casp-1* ^-/-^ murine BMDCs stimulated with heat-killed *T. marneffei* (TM) yeasts (1 MOI) for 18 hr, or LPS plus nigericin as a positive control (n=3). **(B, C)** Representative confocal micrographs and the percentages of ASC specks formation in WT, *Nlrp3^-/-^* murine BMDCs (2x10^6^/ml) stimulated with heat-killed *T. marneffei* (TM) yeasts (1 MOI) for 18 hr, or LPS plus nigericin as a positive control. **(D, E)** Quantitative detection of IL-1β and TNF-α by ELISA in the cell supernatant of WT, *Casp-1* ^-/-^ and *Nlrp3* ^-/-^ murine BMDCs (2x10^6^/ml) stimulated with heat-killed *T. marneffei* (TM) yeasts (1 MOI) for 18 hr with or without LPS priming, or LPS plus nigericin as a positive control. The percentages of ASC speck positive cells are quantified and depicted as mean ± SEM of n=3 mice for each group, and “ND” denotes “not detectable” **(C)**. Data represent mean ± SEM of n=4 mice, and data are analyzed by two-way ANOVA **(D, E)**. *p < 0.05, **p < 0.01, ****p < 0.0001.

To delineate whether IL-1β release induced by *T. marneffei* yeasts was mediated by NLRP3 inflammasome, murine bone marrow-derived DCs (BMDCs) from WT, *Nlrp3* ^-/-^, *Casp1* ^-/-^ mice were co-cultured with heat-killed yeasts, and then NLRP3, caspase-1 p20 and p45 expression were analyzed by immunoblots. As shown in **Figure 4A**, we found that NLRP3 expression was obviously upregulated by yeasts in BMDCs from WT and *Casp-1* ^-/-^ mice. Moreover, pro-caspase-1 (p45) expression in cell lysates was comparable between unstimulated and yeasts-stimulated cells in either WT or *Nlrp3* ^-/-^ mice. Active caspase-1 p20 was detectable in the concentrated supernatant of yeasts-stimulated BMDCs from WT mice, but it was absent in the supernatant from cells deficient in NLRP3 or caspase-1, suggesting that caspase-1 activation is dependent on NLRP3. LPS plus nigericin, which are considered as the activators of classical NLRP3 inflammasome, could activate caspase-1 since active caspase-1 p20 was observed in both cell lysates and supernatant of WT BMDCs.

To determine whether *T. marneffei* yeasts could induce ASC specks to mediate IL-1β processing, BMDCs from WT and *Nlrp3* ^-/-^ mice were co-cultured with yeasts, and the formation of ASC pyroptosomes (“specks”) was examined. Confocal microscopy revealed that ASC pyroptosomes, appearing as a single cytoplasmic speck, was present in WT cells but not in *Nlrp3* ^-/-^ cells stimulated with *T. marneffei* yeasts or LPS plus nigericin (**Figure 4B**). We also quantified the percentage of cells containing ASC specks and observed that around 8% of WT cells formed ASC specks while it was undetectable in *Nlrp3* ^-/-^ cells after *T. marneffei* yeasts stimulation (**Figure 4C**). Similarly, LPS plus nigericin could induce about 29% of WT cells containing ASC specks (**Figure 4C**). Our results indicated that *T. marneffei* yeasts induced ASC speck formation, which was dependent on NLRP3, to induce IL-1β processing.

Next, to further test the hypothesis that IL-1β response to *T. marneffei* yeasts was dependent on NLRP3 inflammasome, BMDCs from WT, *Casp-1^-/-^*, and *Nlrp3* ^-/-^ mice were co-cultured with heat-killed *T. marneffei* yeasts, and IL-1β and TNF-α in the supernatant were examined. As shown in **Figure 4D**, LPS induced a small amount of IL-1β in these BMDCs, whereas there was remarkably lower IL-1β in *Casp-1^-/-^* and *Nlrp3* ^-/-^ BMDCs than WT cells after stimulation with LPS plus nigericin. More importantly, we observed that BMDCs from *Casp-1^-/-^* and *Nlrp3* ^-/-^ mice had significantly impaired IL-1β response to *T. marneffei* yeasts when compared with WT mice (**Figure 4D**). Furthermore, an intriguing observation was that IL-1β production in BMDCs from *Nlrp3* ^-/-^ mice was slightly lower than those from *Casp-1^-/-^* mice after *T. marneffei* yeasts stimulation with or without LPS priming (**Figure 4D**), indicating that IL-1β response to *T. marneffei* yeasts was more dependent on NLRP3 than caspase-1. In contrast to IL-1β production, BMDCs from WT, *Casp-1* ^-/-^, and *Nlrp3* ^-/-^ mice produced comparable levels of TNF-α when co-cultured with *T. marneffei* yeasts (**Figure 4E**). Collectively, these results corroborated that NLRP3 and caspase-1 were required for IL-1β response to *T. marneffei* yeasts in murine BMDCs.

### The NLRP3 Inflammasome Controlled Antifungal Immunity *In Vivo*

**Figure 5:**
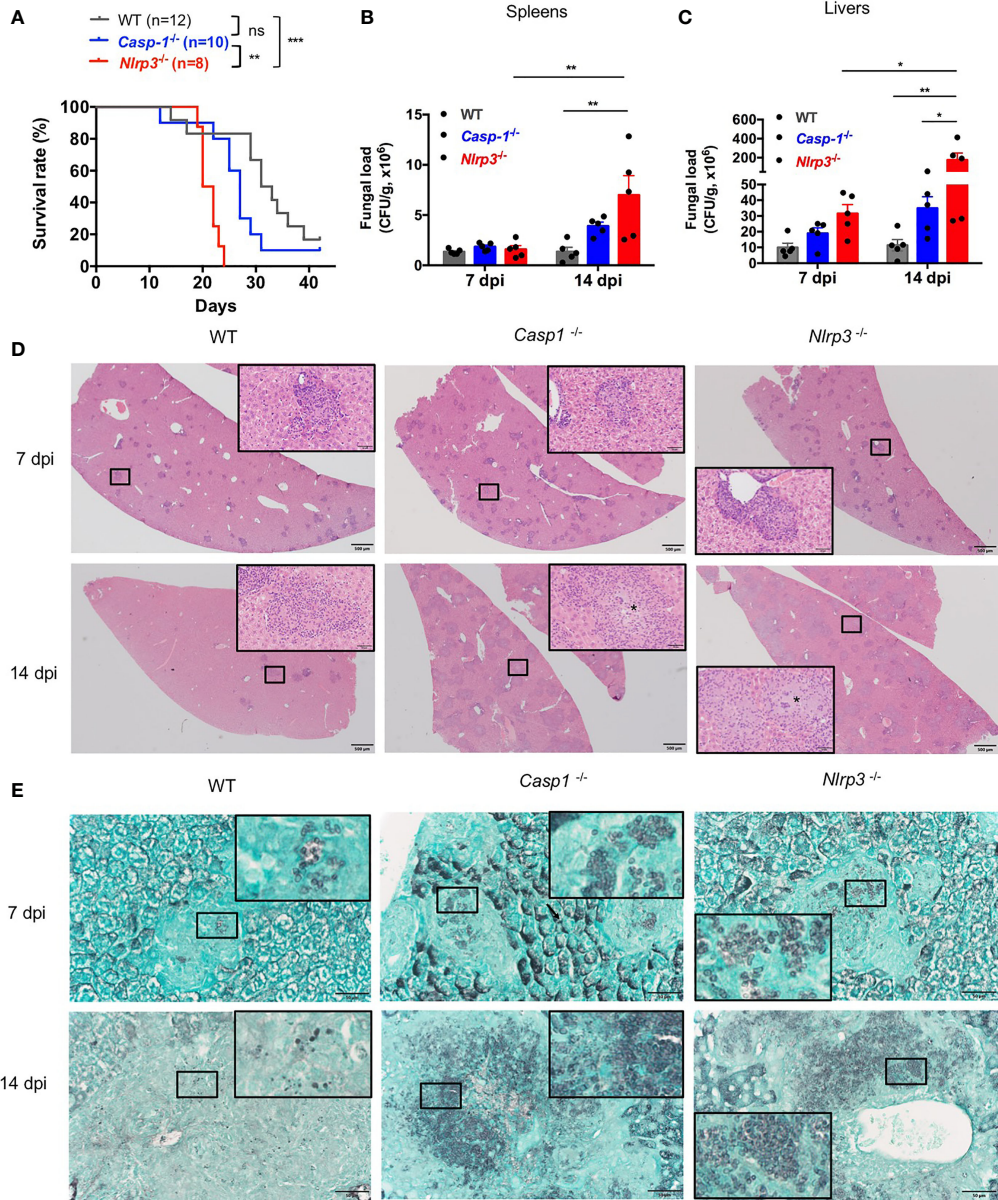
The NLRP3 inflammasome controls antifungal immunity *in vivo*. **(A)** Survival plot of WT, *Casp-1* ^-/-^ and *Nlrp3* ^-/-^ mice intravenously injected with live *T. marneffei* yeasts (5x10^5^ CFU per mouse). **(B, C)** Fungal load analyses of spleens and livers of WT, *Casp-1* ^-/-^ and *Nlrp3* ^-/-^ mice infected with live *T. marneffei* yeasts at 7 and 14 days post infection (dpi). **(D)** Representative HE staining graphs of murine livers of WT, *Casp-1* ^-/-^ and *Nlrp3* ^-/-^ mice infected with live *T. marneffei* yeasts (n=4). Scale bar denotes 500 μm. The insets indicate the magnified area of the smaller box containing granulomas, and the asterisk indicates massive fungal yeasts in the granulomas. **(E)** Representative GMS staining graphs of murine livers of WT, *Casp-1* ^-/-^ and *Nlrp3* ^-/-^ mice infected with live *T. marneffei* yeasts (n=4). Scale bar denotes 50 μm. The insets indicate the magnified area of the smaller box containing *T. marneffei* yeasts. The log-rank tests are performed when compared the survival rates between groups **(A)**. Data are depicted as mean ± SEM of n=5 mice and are analyzed by two-way ANOVA **(B, C)**. ns, not significant. *p < 0.05, **p < 0.01, ***p < 0.001.

To examine the protective role of NLRP3 inflammasome in defense against *T. marneffei* infection *in vivo*, WT, *Casp-1* ^-/-^, *Nlrp3* ^-/-^ mice were infected with 5x10^5^ CFU of *T. marneffei* yeasts per mouse by intravenous injection and their survival was monitored. As shown in **Figure 5A**, *Casp-1* ^-/-^ and *Nlrp3* ^-/-^ mice were more susceptible to *T. marneffei* yeasts infection than WT mice. Of note, *Casp-1* ^-/-^ mice survived longer than *Nlrp3* ^-/-^ mice after fungal infection. These observations suggested that NLRP3 and caspase-1 restrained from *T. marneffei* infection, in particular, NLRP3 had a more important role than caspase-1. To further investigate the factors resulting in the differential survival time among WT, *Casp-1* ^-/-^, *Nlrp3* ^-/-^ mice, murine spleens and livers were collected at 7 and 14 days post infection (dpi) for fungal load detection. There was comparable fungal load in spleens from WT, *Casp-1* ^-/-^, *Nlrp3* ^-/-^ mice at 7 dpi (**Figure 5B**). Nevertheless, significantly elevated fungal load was observed in spleens from *Nlrp3* ^-/-^ mice when compared to WT mice at 14 dpi (**Figure 5B**). Similarly, liver specimen from *Nlrp3* ^-/-^ mice exhibited the highest fungal load, followed by *Casp-1* ^-/-^ mice, and WT mice showed the least fungal load in livers at 7 and 14 dpi (**Figure 5C**). It was worth noting that the fungal load in spleens and livers from WT mice at 14 dpi was similar to that at 7 dpi, whereas the fungal load in these organs from *Nlrp3* ^-/-^ mice at 14 dpi was significantly higher than that 7 dpi (**Figures 5B, C**), indicating that NLRP3 inflammasome played a pivotal role in the control of *T. marneffei* proliferation.

Histological studies of liver sections from WT, *Casp-1* ^-/-^ and *Nlrp3* ^-/-^ mice at 7 dpi showed a great number of granulomas (**Figure 5D**, upper panel). By day 14, granulomas were considerably enlarged and almost replaced the liver parenchyma in *Casp-1* ^-/-^ and *Nlrp3* ^-/-^ mice when compared to those at 7 dpi, but liver sections from WT mice exhibited a smaller number of granulomas (**Figure 5D**, lower panel). HE staining of livers revealed that WT mice had relatively fewer intracellular fungal yeasts within macrophages in the central granulomas than *Casp-1* ^-/-^ and *Nlrp3* ^-/-^ mice (**Figure 5D**, insets of lower panel). Tissue necrosis was not observed in all three strains of mice. GMS staining further verified that fungal yeasts were present in the center of granulomas (**Figure 5E**). Slightly fewer yeasts were observed in WT mice than *Nlrp3* ^-/-^ and *Casp-1* ^-/-^ mice at 7 dpi (**Figure 5E**, upper panel). Importantly, massive number of *T. marneffei* yeasts were seen in the granulomas of *Casp-1* ^-/-^ and *Nlrp3* ^-/-^ livers, while *T. marneffei* yeasts were sparse in the granulomas of WT mice at 14 dpi (**Figure 5E**, lower panel). Spleen sections of *Nlrp3* ^-/-^ mice showed a more severe loss of follicular structure in the white pulp than WT and *Casp*-1^-/-^ mice at 7 dpi (**Figure S3A**, upper panel). No granulomas were observed in the spleens (**Figure S3A**). We did not detect *T. marneffei* in the spleens at 7 dpi, but scattered fungal yeasts could be seen in the spleens of WT, *Nlrp3* ^-/-^ and *Casp-1* ^-/-^ mice at 14 dpi by GMS staining (**Figure S3B**). In conclusion, these histopathological examinations further demonstrated that NLRP3 inflammasome was essential for host defense against *T. marneffei* infection *in vivo*.

### NLRP3 Inflammasome’s Antifungal Immunity Correlates With the Recruitment of CD4 T Cells

**Figure 6:**
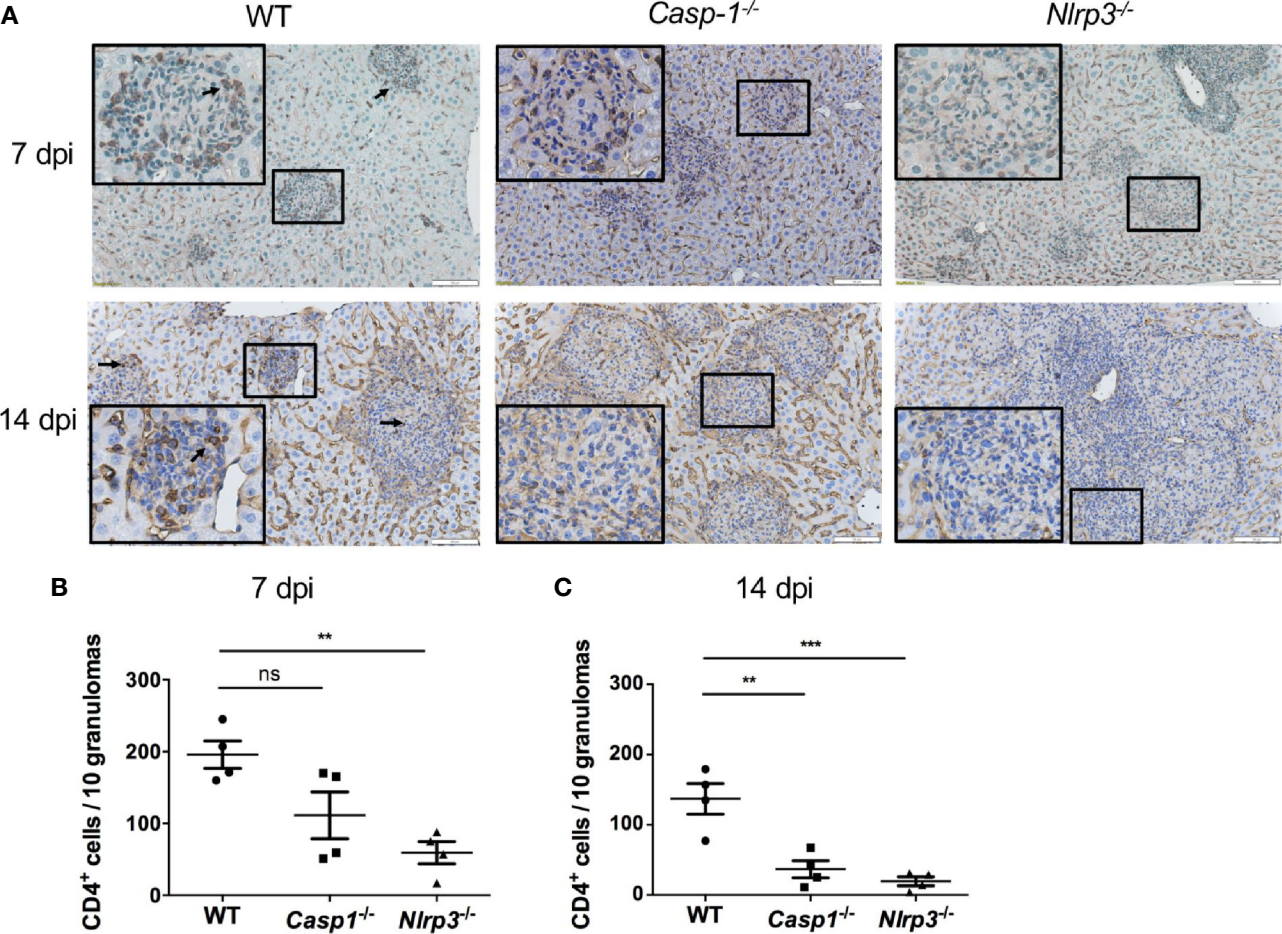
The deficiency of Caspase-1 and NLRP3 undermines the recruitment of CD4 T cells into granulomas in murine liver. **(A)** Representative CD4 staining graphs of murine livers of WT, *Casp-1* ^-/-^ and *Nlrp3* ^-/-^ mice intravenously infected with live *T. marneffei* yeasts (5x10^5^ CFU per mouse) at 7 dpi and 14 dpi. Scale bar denotes 100 μm. The insets indicate the magnified area of the smaller box containing granulomas, and the arrow indicates representative CD4 positive cells in the granulomas. **(B, C)** The number of CD4 positive T cells per 10 granulomas in livers from WT, *Casp-1* ^-/-^ and *Nlrp3* ^-/-^ mice at 7 dpi and 14 dpi, and data represent mean ± SEM of n=4 mice, and they are analyzed by one-way ANOVA. ns, not significant, **p < 0.01, ***p < 0.001.

To investigate the contributing factors to higher mortality and fungal load in *Casp-1* ^-/-^ and *Nlrp3* ^-/-^ mice compared to WT mice, CD4 T cells were stained by immunohistochemistry (IHC) in the livers from *T. marneffei*-infected WT, *Casp-1* ^-/-^, *Nlrp3* ^-/-^ mice. Our results showed that a great number of CD4 T cells were recruited into the surrounding area of granulomas in the livers of WT mice at 7 dpi (**Figures 6A, B**) and 14 dpi (**Figures 6A, C**). By contrast, only a small number of CD4 T cells were observed in the murine livers of *Casp-1* ^-/-^, *Nlrp3* ^-/-^ mice, in particular, at 14 dpi (**Figures 6A–C**). It is also worth noting that the number of CD4 T cells in the granuloma had a downward trend from 7 dpi to 14 dpi among WT, *Casp-1* ^-/-^, *Nlrp3* ^-/-^ mice (**Figures 6B, C**), implying that the relative abundance of T cells was reduced with the proliferation of *T. marneffei*.

## Discussion

*T. marneffei* is an important form of endemic mycoses causing major morbidity and mortality in patients with HIV infection as well as those with primary and secondary immunodeficiencies. However, innate immune response against *T. marneffei* is largely unknown. For the first time, our study characterized Dectin-1 and Syk mediated IL-1β response towards *T. marneffei*, and NLRP3-ASC-caspase-1 inflammasome was activated by these microbes. Importantly, our murine model of disseminated talaromycosis demonstrated that, compared to wild-type mice, *Casp-1* ^-/-^, *Nlrp3* ^-/-^ mice displayed higher mortality rate and fungal load, which correlated with impaired CD4 T cell recruitment into granulomas.

Our findings reveal differential cytokine responses towards *T. marneffei* conidia and yeasts in human PBMCs (**Figures 1A–D**). These interesting findings indicate that conidia are less immunogenic than yeasts, as shown by negligible IL-1β and TNF-α production in PBMCs during the first 24-48 hours of co-culture. The delayed rise in TNF-α, and IL-1β to a lesser extent, after 3 days of co-culture, probably represents the switch of conidia to yeast form *in vitro*, suggesting that the transformation of conidia into yeasts would be required for IL-1β response. It is interesting to note that our observation that *T. marneffei* yeasts alone induced mature IL-b production in human PBMCs and monocytes rather than macrophages (**Figure 2**) matches what was previously found in these cells stimulated with single LPS (23).

Blockade of Dectin-1 with neutralizing antibodies suppressed the production of IL-1β in response to *T. marneffei* yeasts in human CD14^+^ monocytes. This suggests that Dectin-1 plays a role in initiating downstream signaling that leads to pro-IL-1β production, which has been demonstrated in other fungal pathogens such as *C. albicans*, *H. capsulatum* and *A. fumigatus* (17, 26, 27). Acquired immunity to other dimorphic fungi such as *Blastomyces dermatitidis, Histoplasma capsulatum* and *Coccidioides posadasii* infection variably depends on innate sensing by Dectin-1, Dectin-2 and Mincle (28), and the role of these CLRs receptors in cytokine response against *T. marneffei* will require further studies. Furthermore, *T. marneffei* yeasts induced Syk phosphorylation and blocking Syk activity markedly impaired pro-IL-1β and mature IL-1β production (**Figures 3E–G**). Since CARD9 is an adaptor protein of downstream of Dectin-1/Syk signaling pathway (25), our data implicate that IL-β production in response to *T. marneffei* yeasts might be CARD9-dependent, although we could not exclude the possibility that CARD9-independent RAS-RAF1 signaling pathway (24) might also participate in IL-1β production.

TLR2 and Dectin-1 physically associate with one another and synergize to augment anti-fungal response by modulating cytokine production (17, 29, 30). Our results showed that blockade of TLR2 led to increased IL-1β production in *T. marneffei* yeasts-stimulated human monocytes (**Figure 3C**), suggesting that TLR2 ligation might exert a negative effect on IL-1β-mediated immune response against *T. marneffei.* TLR2-deficient mice infected with *P. brasiliensis* have preferential activation of Th17 response and lower fungal load, while Treg expansion is diminished and aggravates lung inflammation (31). A similar role of TLR2 was also observed in a murine model of disseminated candidiasis, where TLR2 exerts anti-inflammatory effect by promoting IL-10 production and Treg cell proliferation (32).

NLRP3 inflammasome activation has been demonstrated to contribute to host defense against diverse fungal infections, including *C. albicans, A. fumigatus and P. brasiliensis*, *H. capsulatum, S. schenckii* infection (17, 18, 26, 33, 34). Consistent with these observations, our study defines the critical role of NLRP3 inflammasome activation in controlling *T. marneffei* proliferation *in vivo*. An intriguing observation is that the significantly shorter survival time of *Nlrp3* ^-/-^ mice correlates with higher fungal load, particularly in the liver at 14 dpi, compared with *Casp-1* ^-/-^ mice (**Figures 5A–C**). These *in vivo* data also correlate well with IL-1β response to *T. marneffei* in murine BMDCs *in vitro*, reinforcing the critical role of IL-1β in protective immunity against *T. marneffei.* It is interesting to note that there was the residual IL-1β response to *T. marneffei* in BMDCs from *Casp-1* ^-/-^ and *Nlrp3* ^-/-^ mice (**Figure 4D**), and particularly the lower fungal load and better survival in *Casp-1* ^-/-^ mice compared to *Nlrp3* ^-/-^ mice (**Figures 5A–C**), indicating that in addition to caspase-1, there is an alternative caspase molecule to be implicated in the NLRP3 inflammasome activation in response to *T. marneffei*. That molecule could be caspase-8 since it has been suggested to activate non-canonical inflammasome (35).

Inflammasomes that are key components of the innate immune responses can shape adaptive immune responses against pathogens through the release of inflammatory cytokines (IL-1β and IL-18) and pyroptotic cell-derived antigens (20, 36). Inflammasomes-derived IL-1β and IL-18 favor Th17 and Th1 cell differentiation, respectively, pertaining to CD4 T cell responses (20, 36). These events have been reported in host protection against fungal infections, such as *C. albicans* (19), *P. brasiliensis* (37, 38). In this study, we attempted to dissect the link between *T. marneffei*-induced NLRP3 inflammasome activation and CD4 T cell responses upon fungal exposure. Our data showed that diminished recruitment of CD4 T cells in the liver of *Casp-1* ^-/-^ and *Nlrp3* ^-/-^ mice correlates with their higher mortality and fungal load when compared to WT mice, implicating that CD4 T cells are important for the activated NLRP3 inflammasome to control *T. marneffei* infection; however, we failed to detect a significant difference in the production of murine IFN-γ and IL-17A in spleens between WT and knockout mice (data not shown). These scenarios could ascribe to the possibility that NLRP3 inflammasomes instruct the trafficking and recruitment of effector CD4 T cells into the site of fungal infection (livers) while having a limited effect on CD4 T cell priming and differentiation in spleens *in vivo*. In addition, to better understand the innate instruction of CD4 T cells by NLRP3 inflammasome, we may need to characterize the phenotype of these infiltrated CD4 T cells in the murine livers in the future. In human cells, caspase-1 inhibition significantly attenuates IFN-γ and IL-17A production *in vitro*, reflecting the notion that activated NLRP3 inflammasome could promote Th1 and Th17 immune responses towards *T. marneffei* infection.

It has been demonstrated that Th1 and Th17 immune responses are essential for host defense against fungal infection (39–43). T cells are also considered important to defend against *T. marneffei* infection since athymic mice show fatal mycosis while fungus is better controlled in euthymic hosts after *T. marneffei* pulmonary infection (44). Moreover, up-regulation of Th1 related cytokines, IFN-γ and IL-12, was observed in the spleens, and all wild type mice survived with a self-limiting infection whereas IFN-γ-deficient mice died at day 18 post infection, when BALB/c mice were inoculated with 3x10^5^ *T. marneffei* conidia (45). In comparison, our current systemic talaromycosis model with injection of 5x10^5^ *T. marneffei* yeasts into mice with C57BL/6 background showed that 10 out of 12 wild type mice eventually succumbed to fungal infection in spite of significantly prolonged survival time compared to *Nlrp3* ^-/-^ mice, implying that establishment of *T. marneffei* infection model at a lower dose that at least half of wild type mice survive would be more convinced to demonstrate the important role of NLRP3 in defense against its infection in the following study. In addition, Sisto F et al. (2003) also revealed that wild type mice at 7 dpi had the comparable fungal load with those mice at 14 dpi, but considerable increase of fungal load was seen in IFN-γ knockout mice at 14 dpi compared to 7 dpi, in both spleens and livers (45). Similar phenotype was also observed in the current study of wild type and *Nlrp3* ^-/-^ mice, indicating both IFN-γ and NLRP3 have protective roles after *T. marneffei* infection. However, comparison between the antifungal effects of IFN-γ and NLRP3 needs to use the infection model with the same mouse background and infection dose.

Clinically, compromised function of CD4 T cells was found to predispose to disseminated talaromycosis, such as HIV-positive patients with severe T cell lymphopenia (CD4^+^ T cell count < 100/μl blood) (1, 6), and HIV-negative patients with autoantibodies against IFN-γ, CD40 ligand deficiency, and autosomal dominant (AD) hyper-IgE syndrome that primarily involve the defect in Th1 or/and Th17 immune responses (6, 7, 46). Nevertheless, the role of CD4 T cells, particularly Th17 cells, in the antifungal immunity to *T. marneffei* still needs to be further investigated by using IL-17A knockout mice infection models. Another limitation of this study is that our experimental design involves a murine systemic talaromycosis model which represents the advanced stage of *T. marneffei* infection; how conidia-yeasts transition in the airway epithelium impacts on the activation of NLRP3 inflammasome and induction of adaptive T-cell response at the earlier stage of infection requires animal studies using intranasal instillation technique for delivery of *T. marneffei* conidia.

This study enhances our understanding of host defense mechanisms against *T. marneffei*. Knowledge about human immune response towards *T. marneffei* will have therapeutic implications in managing patients suffering from this fatal infection. Delineation of the roles of various cytokines towards protection against *T. marneffei* will provide important information to the treatment of patients who have secondary immunodeficiency resulting from the use of biologics for immunological disorders, for example, with the increasingly wide clinical use of IL-1 receptor antagonist (e.g. Anakinra, Rilonacept) or neutralization antibodies (Canakinumab) for patients with auto-inflammatory or auto-immune diseases, and therefore clinicians should be alerted to the susceptibility and signs of talaromycosis when treating patients who reside in endemic regions where there is increased risk of environmental exposure to *T. marneffei*.

To conclude, in this study, we first demonstrate that *T. marneffei* yeasts rather than conidia induce IL-1β response, which is differentially regulated in distinctive cell types. Our findings show that Dectin-1/Syk signaling pathway mediates pro-IL-1β production after microorganism stimulation. *T. marneffei* yeasts also trigger the assembly of NLRP3-ASC-caspase-1 inflammasome to facilitate IL-1β maturation *in vitro*. *Nlrp3* ^-/-^, *Casp1* ^-/-^ mice are more susceptible to *T. marneffei* yeasts with higher fungal load and fewer recruited CD4 T cells than wild-type mice. Collectively, our study highlights the importance of NLRP3 inflammasome in host defense against *T. marneffei* infection and sheds new light on the interaction between host and fungal pathogens.

## Data Availability Statement

The original contributions presented in the study are included in the article/**Supplementary Material**. Further inquiries can be directed to the corresponding author.

## Ethics Statement

The studies involving human participants were reviewed and approved by Institutional Review Boards of the University of Hong Kong/Hospital Authority Hong Kong West Cluster. The patients/participants provided their written informed consent to participate in this study. The animal study was reviewed and approved by the Committee on the Use of Live Animals in Teaching and Research (CULATR) at the University of Hong Kong.

## Author Contributions

Author contributions were as follows: HM, JC, Y-LL, PW, and PL designed the experiments and analyzed the results. HM, YT, LK, C-CT, and SP performed the experiments. HM and PL wrote and revised the manuscript. All authors contributed to the article and approved the submitted version.

## Funding

This work was supported by the Edward and Yolanda Wong Fund, the RGC General Research Fund (GRF) (HKU project code: 17111814), and the Health and Medical Research Fund [No. HKM-15-M07 (commissioned project)].

## Conflict of Interest

The authors declare that the research was conducted in the absence of any commercial or financial relationships that could be construed as a potential conflict of interest.

## Publisher’s Note

All claims expressed in this article are solely those of the authors and do not necessarily represent those of their affiliated organizations, or those of the publisher, the editors and the reviewers. Any product that may be evaluated in this article, or claim that may be made by its manufacturer, is not guaranteed or endorsed by the publisher.
