# Supplementary material for: NLRP3 Inflammasome Contributes to Host Defense Against Talaromyces marneffei Infection
Source: Front Immunol. 2021 Nov 29;12:760095. doi: 10.3389/fimmu.2021.760095 (PMC8666893; doi:10.3389/fimmu.2021.760095)
Supplement: Supplementary file 1 [file DataSheet_1.docx]

**Supplementary Material**


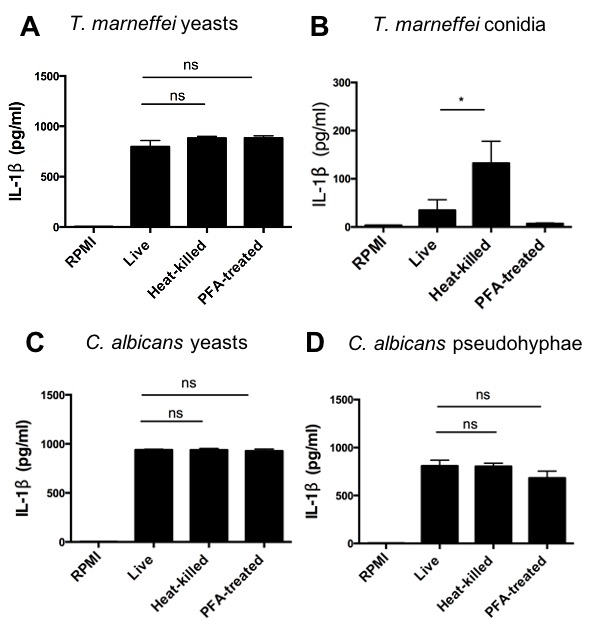


**FIGURE S1 The viability of *T. marneffei* yeasts is dispensable for IL-1β response in human PBMCs**. (A and B) Quantification of IL-1β by ELISA in the cell supernatant of human PBMCs (4x10^6^/ml) stimulated with live, heat-killed or PFA-treated *T. marneffei* yeasts (0.5 MOI) and conidia (0.5MOI) (n=5) for 18 hr. (C and D) Quantification of IL-1β by ELISA in the cell supernatant of human PBMCs (4x10^6^/ml) stimulated with live, heat-killed or PFA-treated *C. albicans* yeasts (0.5 MOI) and pseudohyphae (0.5MOI) (n=5) for 18 hr. Data are depicted as mean ± SEM, and are analyzed by one-way ANOVA. ns =not significant, *p<0.05.

**FIGURE S2** **Elevation of syk phosphorylation induced by *T. marneffei* and reduction of IFN-γ and IL-17A production by caspase-1 inhibitor.** (A) Representative cytometric graph of phospho-Syk in human CD14^+^ monocytes (2x10^6^/ml) stimulated with heat-killed *T. marneffei* (TM) yeasts (0.5 MOI) for 18 hr (n=3, mean ± SEM) (B) Human PBMCs were co-cultured with heat-killed *T. marneffei* yeasts for 5 days in the presence or absence of caspase-1 inhibitor (Z-YVAD), and IFN-γ and IL-17A in the supernatant was measured by ELISA. Data are depicted as mean ± SEM, and are analyzed by paired t test. ***p<0.001


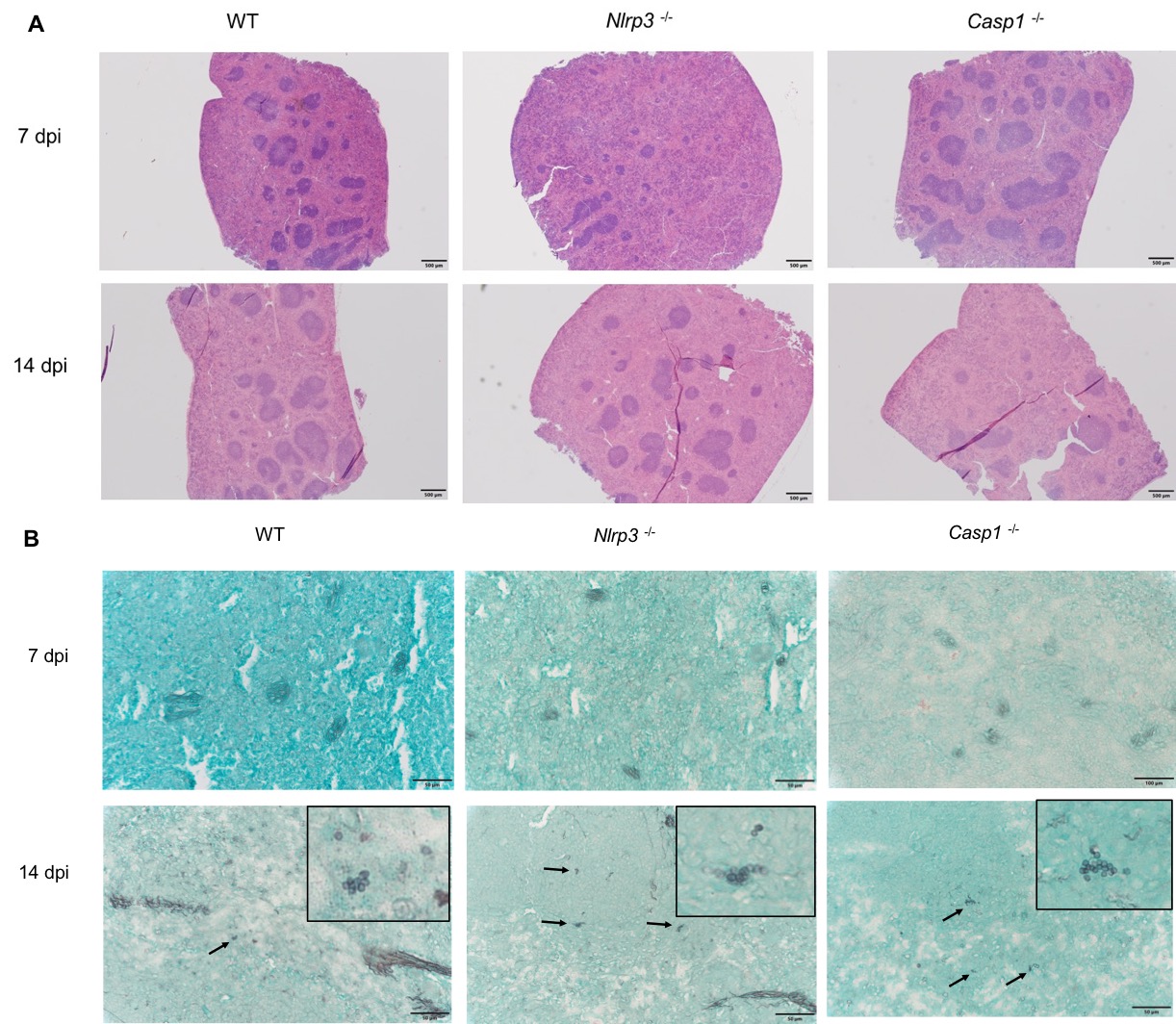


**FIGURE S3** **Histopathological analysis of murine spleens.** (A) Representative sections of HE staining of spleens from WT, *Nlrp3*^-/-^ and *Casp-1*^-/-^ mice intravenously infected *T. marneffei* yeasts (5x10^5^ CFU per mouse) at 7 days and 14 days post infection (dpi) (n=4). Scale bar denotes 500 μm. (B) Representative graphs of GMS staining of spleens from WT, *Nlrp3*^-/-^ and *Casp-1*^-/-^ mice intravenously infected *T. marneffei* yeasts (5x10^5^ CFU per mouse) at 7 days and 14 days post infection (dpi) (n=4). Scale bar denotes 50 μm. Arrows indicate *T. marneffei* yeasts, and the insets indicate the magnified areas containing *T. marneffei* yeasts.
